# Renal Ischemia–Reperfusion and Uremic Toxins Modulate the Aortic Adenosinergic Axis in Acute Kidney Injury

**DOI:** 10.3390/ijms27146296

**Published:** 2026-07-15

**Authors:** Ana Carolina da Costa Peres, Jackeline Rodrigues Ramos, Jeferson Stabile, Carmen Lucia Sanz Alarta, Lia Sumie Nakao, Fernanda Tibolla Viero, Henning Ulrich, Cristina Ribas Fürstenau

**Affiliations:** 1 Laboratory of Vascular Biochemistry, Center for Natural and Human Sciences, Federal University of ABC, Santo André 09280-560, São Paulo, Brazil; ana.peres@aluno.ufabc.edu.br (A.C.d.C.P.); jackeline.ramos@aluno.ufabc.edu.br (J.R.R.); jefersonstabile@gmail.com (J.S.); 2 Department of Basic Pathology, Biological Sciences Sector, Federal University of Paraná, Curitiba 81531-980, Paraná, Brazil; lusanz.alarta@gmail.com (C.L.S.A.); lia.nakao@ufpr.br (L.S.N.); 3 Department of Biochemistry, Institute of Chemistry, University of São Paulo, São Paulo 05508-000, Brazil; fetibollaviero@gmail.com (F.T.V.); henning@iq.usp.br (H.U.)

**Keywords:** indoxyl sulfate, kidney-vessel communication, adenosine, aorta, inflammation

## Abstract

Acute kidney injury (AKI) is characterized by a rapid decline or sudden loss of renal function over hours to days. Pathophysiological triggers such as renal ischemia–reperfusion (IR) injury and the accumulation of uremic toxins (UTs), notably indoxyl sulfate (IS), can initiate AKI and affect vascular beds distant from the ischemic site, like the aorta. In this context, purinergic signaling becomes relevant, since its components regulate vascular tone and inflammatory responses. This study aimed to evaluate the impact of AKI induced by IR with or without IS administration on purinergic signaling in the aorta of mice. Renal ischemia was induced by the occlusion of the left renal pedicle for 60 min, followed by reperfusion for 8 days (IR 8) or 15 days (IR 15). Some animals were also treated with saline solution or IS for 15 days. The IR15 group exhibited increased plasma IS concentrations and upregulated adenosine receptor gene expression. Furthermore, in the IR+IS group, there was increased expression of A1, A2a, NTPDase 1, and 2. This shift toward an adenosine-enriched signaling environment may represent a key mechanism linking renal injury to systemic vascular inflammation.

## 1. Introduction

In recent decades, the incidence of acute kidney injury (AKI) has increased considerably. Given the clinical outcomes observed, particularly in the long term, AKI has become a growing global health concern [1]. It is a frequent complication among patients admitted to intensive care units (ICUs), with an incidence ranging from 5% to over 10%, and is associated with high morbidity and mortality rates [1,2]. The Kidney Disease: Improving Global Outcomes (KDIGO) clinical practice guidelines define criteria for the diagnosis, classification, prevention, and treatment of AKI. To establish a unified framework, the previous RIFLE and AKIN criteria were consolidated into a three-stage classification based on serum creatinine levels and urine output [3]. Moreover, the causes of AKI are highly heterogeneous and can be categorized according to their underlying pathophysiological mechanisms. In addition, patient history and clinical examination remain fundamental for determining the etiology of the condition [4].

In hospital settings, ischemic kidney injuries, whether generalized or localized, resulting from surgery, sepsis, or trauma account for a substantial proportion of AKI cases. Ischemia–reperfusion (IR) injury arises from an insufficient supply of oxygen and nutrients, coupled with reduced clearance of metabolites by renal cells [2,5]. This imbalance between oxygen delivery and tissue demand leads to the accumulation of metabolic waste products, causing tubular epithelial cell damage and nephrotoxicity, which in turn promotes the buildup of uremic toxins such as indoxyl sulfate (IS) [4].

Indoxyl sulfate (IS) is a low–molecular-weight uremic toxin derived from the metabolism of the amino acid tryptophan by the intestinal microbiota [6,7]. Although its free form has a low molecular weight, IS binds strongly to albumin in the bloodstream, which markedly limits its removal by dialysis [8]. Elevated IS concentrations stimulate the proliferation of vascular smooth muscle cells (VSMCs) in rats and enhance the generation of reactive oxygen species (ROS) [9,10], leading to damage in the kidneys, heart, and blood vessels. These effects contribute to systemic inflammation and favor the progression from acute kidney injury (AKI) to chronic kidney disease (CKD) [6].

The interplay between the kidney and the vascular system is essential for maintaining homeostasis. Renal injury, a major cause of acute kidney injury (AKI), increases the production of reactive oxygen species (ROS), leading to reduced nitric oxide synthesis—an important vasodilator. This reduction contributes to endothelial dysfunction not only in renal vessels but also in those of other organs, such as the heart [11]. Moreover, studies have shown that prolonged ischemia (~1 h) followed by reperfusion results in extensive necrosis and apoptosis, destroying the proximal tubules of the outer medulla. This process also triggers the release of molecules such as ATP, a damage-associated molecular pattern (DAMP), from the intracellular to the extracellular environment [12,13,14].

Purinergic signaling, first proposed more than 40 years ago by Geoffrey Burnstock, is a cellular communication system involving effectors (nucleotides and nucleosides), receptors (purinoceptors), and enzymes (ectonucleotidases). Extracellular purines (ATP, ADP, and adenosine) and pyrimidines (UDP and UTP) act as key signaling molecules that mediate diverse biological effects through cell-surface purinergic receptors [15]. Two major families of purinergic receptors are recognized: the P1 family of G protein-coupled, adenosine-responsive receptors—subdivided into A1, A2A, A2B, and A3—and the P2 family, which includes P2X ATP-gated ion channels and P2Y metabotropic receptors [15]. The purinergic system also comprises enzymes responsible for the hydrolysis of extracellular nucleotides and nucleosides, collectively known as ectonucleotidases. These enzymes are grouped into four gene families: ectonucleotide pyrophosphatase/phosphodiesterases (ENPPs), alkaline phosphatases, ectonucleoside triphosphate diphosphohydrolases (ENTPDases), and ecto-5′-nucleotidase [16].

The role of purinergic signaling in the vasculature has become increasingly evident. Depending on the cell type and the receptor activated, purines can exert opposite effects, such as vasoconstriction or vasodilation [17]. Purinergic signaling also plays a significant role in vascular inflammation, as extracellular ATP acts as a ubiquitous damage-associated molecular pattern (DAMP) and, consequently, as a key inflammatory mediator. Within blood vessels, three major ectonucleotidases are expressed: NTPDase1 (CD39), NTPDase2, and ecto-5′-nucleotidase (CD73). NTPDase1 exhibits high affinity for ATP and hydrolyzes both ATP and ADP with comparable efficiency. Thus, despite its greater affinity for ATP, its catalytic activity toward ADP remains substantial, resulting in nearly proportional hydrolysis of both substrates. In contrast, NTPDase2 preferentially hydrolyzes ATP and displays low activity toward ADP. NTPDase1, therefore, not only binds ATP with high affinity but also converts ADP to AMP at similar rates. Both enzymes are expressed in endothelial cells and vascular tissues [16]. Together, these two enzyme subtypes form the CD39–adenosinergic axis, which regulates the balance between extracellular ATP and adenosine, thereby controlling their receptor binding and downstream signaling actions [18].

In a recent study by our research group, we demonstrated that renal ischemia–reperfusion injury alters purinergic signaling in the aorta of mice. Gene expression levels of P2Y1, P2Y2, P2Y6, and P2X4 receptors increased 15 days after ischemic surgery, along with the ectonucleotidases NTPDase2 and ecto-5′-nucleotidase. These findings indicate that renal ischemia-induced AKI can modulate components of purinergic signaling in vascular beds distant from the ischemic site [19]. Furthermore, enhanced ectonucleotidase activity may reflect increased adenosine production and, consequently, upregulation of adenosine receptor expression.

The incidence of AKI has risen markedly in recent decades, and several studies have shown that AKI leads to the accumulation of metabolites such as indoxyl sulfate (IS), which, in severe cases, can exert systemic toxicity [4]. It is also well established that under inflammatory and hypoxic conditions, adenosine acts as a protective molecule and serves as an important modulator of vascular function through adenosinergic receptors [20]. However, studies investigating the effects of ischemia–reperfusion-induced AKI, with or without IS exposure, on vascular beds distant from the kidney—such as the aorta—remain scarce, particularly regarding the adenosinergic axis. Therefore, the present study focuses on the effects of AKI induced by IR, with or without IS treatment, on the aorta of mice, and explores how purinergic signaling contributes to this inflammatory process.

## 2. Results

### 2.1. AKI by Renal IR and IR+IS Causes Kidney Trophism

After ischemia–reperfusion (IR), the left kidney exhibited a whitish appearance and reduced mass, likely due to cell loss, fibrosis, and collagen deposition (Figure S1). A significant 35.8% decrease in the left kidney weight of IR15 group animals was observed compared with the Sham and IR8 groups, indicating organ atrophy. In the same group, a 16.2% increase in the right kidney weight was also detected relative to the control group, suggesting compensatory hypertrophy of the contralateral organ (Figure S1A–D). This hypertrophy may result from an increase in nephron size and functional adaptation to compensate for the injury sustained by the left kidney, as well as an attempt to restore the glomerular filtration rate affected by the ischemic process [21].

Uremic toxins are known to accumulate in various organs and tissues of AKI patients, promoting free radical generation, damaging renal tubular cells, and contributing to nephrotoxicity. Therefore, in addition to the Sham, IR8, and IR15 groups previously discussed, morphometric data were also analyzed for animals treated with IS for 15 days following IR surgery (Figure S1E–G). In this experimental set, a significant reduction of 29% and 36% in left kidney mass was observed in the IR + Saline and IR + IS groups, respectively, compared with their corresponding Sham groups, confirming organ atrophy (Figure S1G). As expected, right kidney mass increased by 11% and 15% in the IR groups relative to their respective Sham controls, demonstrating compensatory hypertrophy in these animals (Figure S1F).

Acute kidney injury (AKI) has long been evaluated based on serum and plasma levels of creatinine and urea, as these markers directly reflect changes in the glomerular filtration rate (GFR) [22]. Urea and creatinine levels did not differ between animals in the IR15 group and those in the Sham group (Figure S2A,B). We also assessed creatinine and urea levels in animals subjected to IS treatment following IR surgery (Figure S2C,D). Although a slight increase in urea levels was observed in the IR + Saline group compared with the control group, no significant differences in creatinine levels were detected in this group. Furthermore, plasma urea and creatinine concentrations remained unchanged in animals exposed to IS compared with the other groups. While urea and creatinine have served as conventional biomarkers for AKI assessment, their diagnostic utility is limited by poor sensitivity and specificity, prompting the adoption of newer and more precise indicators, as discussed later.

### 2.2. IR Surgery Induces the Accumulation of Uremic Toxins in Mice

Although no changes were detected in urea and creatinine levels—and despite ongoing discussions regarding the most reliable biomarkers for AKI—plasma concentrations of other uremic toxins, including indoxyl sulfate (IS), p-cresyl sulfate (PCS), and indoleacetic acid (IAA), were measured. Unlike urea and creatinine, which are small and water-soluble molecules, these toxins are predominantly bound to plasma proteins, a characteristic that hinders their filtration through the glomerular barrier and leads to their accumulation under pathological conditions [7,23].

**Figure 1:**
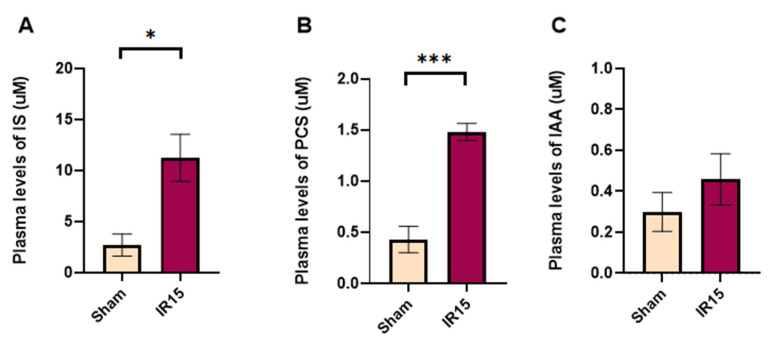
Plasma levels of uremic toxins IS (**A**), PCS (**B**), and IAA (**C**) of Sham and IR15 animals (n = 5 animals per group) (* *p* < 0.05, *** *p* < 0.001). IS = Indoxyl Sulfate; PCS = p-cresyl sulfate, and IAA = Indoleacetic Acid.

The results revealed a 315% and 243% increase in plasma concentrations of indoxyl sulfate (IS) and p-cresyl sulfate (PCS), respectively, in the IR15 group compared with the Sham group, suggesting impaired renal filtration and consequent accumulation of these toxins (Figure 1).

### 2.3. AKI by Renal IR and IR+IS Increases the Gene Expression of All Adenosine Receptors in the Mouse Aorta

A previous study from our research group demonstrated increased transcript levels of purinergic P2Y1, P2Y2, P2Y6, and P2X4 receptors, along with elevated gene expression of NTPDase2 and ecto-5′-nucleotidase, as well as enhanced hydrolysis of ATP and AMP in the aorta of IR15 animals [19]. These findings likely indicate a shift in this signaling pathway toward the adenosinergic axis, promoting greater adenosine production and increased activation of P1 receptors. Therefore, in the present study, we evaluated the gene expression of adenosine receptors and ectonucleotidases in the aorta of animals from all experimental groups.

**Figure 2:**
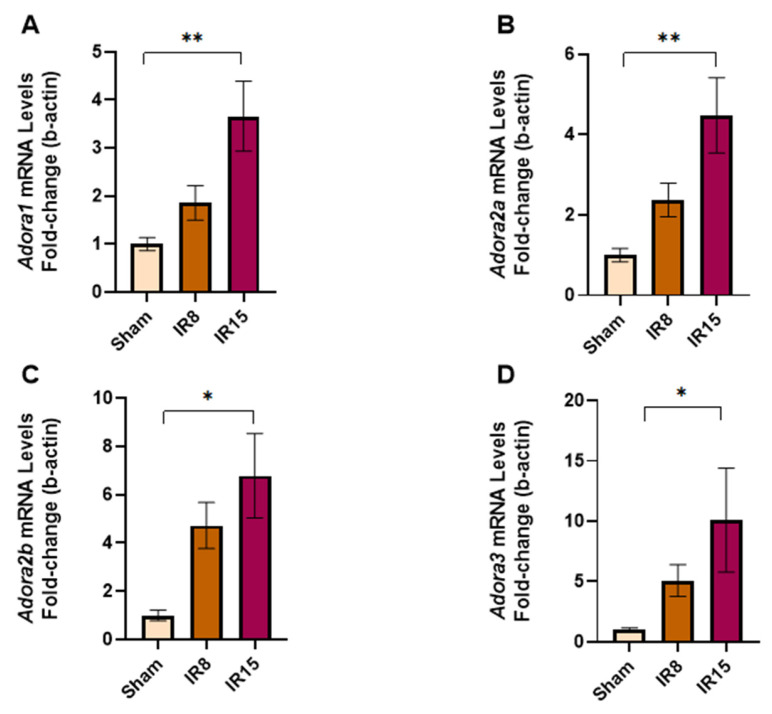
Upregulation of adenosine receptor expression after renal IR injury. Relative gene expression of A1 (**A**), A2a (**B**), A2b (**C**), and A3 (**D**) receptors in aortas of the Sham, IR8, and IR15 groups (n = 5 animals per group). Β-actin gene expression, which did not vary under the chosen experimental conditions, was used for normalization and calculation of relative gene expression (** *p* < 0.01, * *p* < 0.05).

As expected, gene expression levels of all P1 receptors in the aorta of IR15 animals were higher than those in the Sham group (Figure 2), potentially reflecting greater activation of these receptors in IR-induced AKI.

Similarly to what was observed in the ischemia–reperfusion (IR)-induced AKI model, the gene expression of several adenosinergic receptors was also altered in the IR model combined with IS exposure, underscoring the relevance of these receptors in modulating the vascular response.

**Figure 3:**
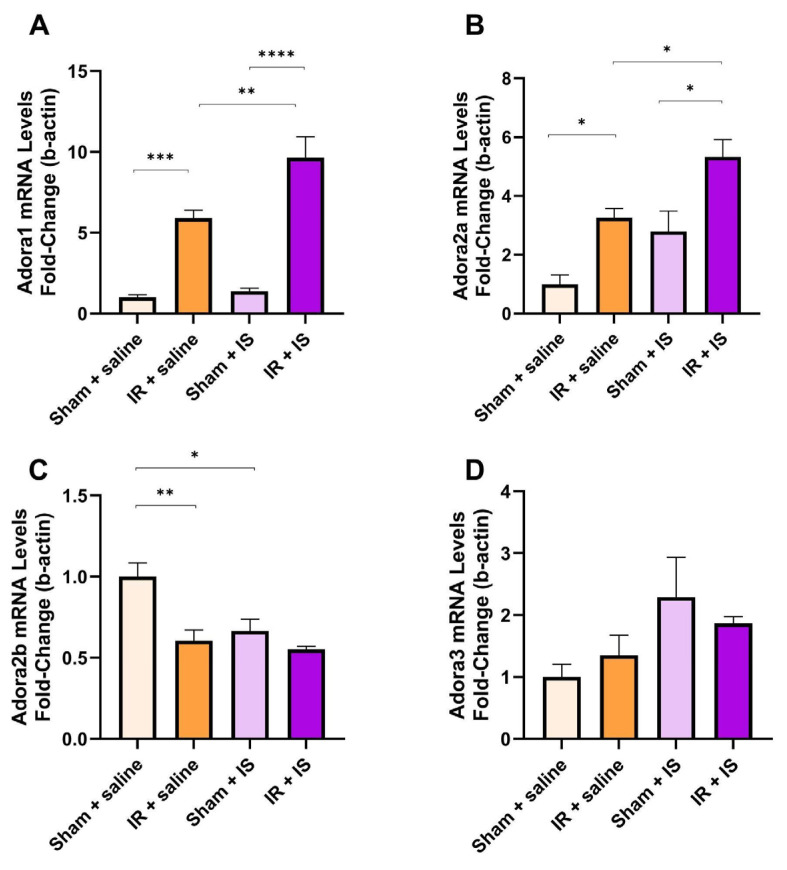
Differential expression of adenosine receptor after renal IR+IS injury. Relative gene expression of A1 (**A**), A2a (**B**), A2b (**C**), and A3 (**D**) receptors in aortas of animals of the Sham+Saline, IR+Saline, Sham+IS, and IR+IS groups (n = 5 animals per group). Β-actin gene expression, which did not vary under the chosen experimental conditions, was used for normalization and calculation of relative gene expression (**** *p* < 0.0001, *** *p* < 0.001, ** *p* < 0.01, * *p* < 0.05).

As shown in Figure 3, transcript levels of the A1 receptor were significantly increased—approximately 491% and 597%—in the aorta of IR and IR + IS animals, respectively, compared with their corresponding controls, and by 63% in IR compared with IR + IS animals (Figure 3A). Similarly, A2A receptor transcripts increased by approximately 226% and 91% in the same groups, and by 63% in IR compared with IR + IS animals (Figure 3B). In contrast, gene expression of the A2B receptor was significantly decreased in the IR + Saline and Sham + IS groups compared with the Sham + Saline group (Figure 3C). No changes were observed in A3 receptor transcript levels in the aortas of any experimental group (Figure 3D).

### 2.4. Renal IR Injury Associated with IS Treatment Modulates Ectonucleotidases and ADA Gene Expression in the Aorta of Mice

**Figure 4:**
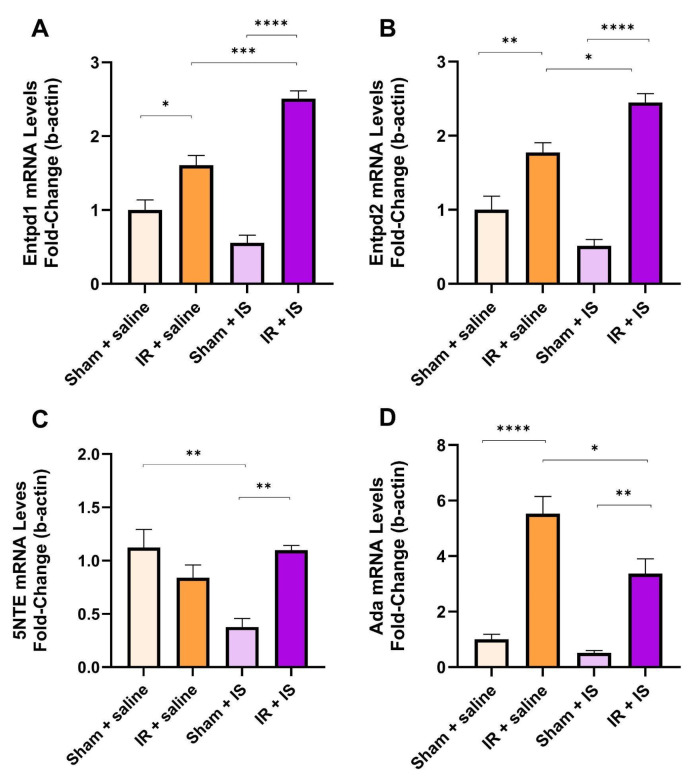
Ectonucleotidases and adenosine deaminase (ADA) gene expression in aorta after renal IR injury plus IS administration. Relative gene expression of NTPDase1 (**A**), NTPDase2 (**B**), Ecto-5’-nucleotidase (**C**) and ADA (**D**) in aortas of animals of Sham+Saline, IR+Saline, Sham+IS, and IR+IS groups (N = 5 animals per group). Β-actin gene expression, which did not vary under the chosen experimental conditions, was used for normalization and calculation of relative gene expression (**** *p* < 0.0001, *** *p* < 0.001, ** *p* < 0.01, * *p* < 0.05).

In addition to the gene expression analysis of P1 receptors, the expression of ectonucleotidases was also evaluated in the aorta of mice from all experimental groups. Expression levels of NTPDase2 and ecto-5′-nucleotidase were increased in the aorta of IR15 animals, as previously reported by Stabile and collaborators [19]. Therefore, in the present study, the analysis of these enzymes focused on the IR model combined with IS administration (Figure 4).

Our results show increased expression of NTPDase1 and NTPDase2 in the IR groups compared with their respective Sham controls (Figure 4A,B). A significant rise of 56% and 38% in NTPDase1 and NTPDase2 expression, respectively, was also observed between the IR + Saline and IR + IS groups. These findings suggest that following IR injury and IS administration, NTPDase1 and NTPDase2 may regulate extracellular ATP levels and, consequently, redirect purinergic signaling toward adenosine production.

Gene expression of ecto-5′-nucleotidase was decreased in Sham + IS animals compared with their Sham + Saline counterparts (Figure 4C), indicating that IS may directly alter the purinergic pathway in the aorta, even in the absence of ischemic injury. However, in the aorta of IR + IS animals compared with Sham + IS animals, a restoration of basal enzyme levels was observed.

In addition to the enzymes that convert ATP into adenosine, another key enzyme in this pathway is adenosine deaminase (ADA), which catalyzes the deamination of adenosine to inosine [24]. Also known as adenosine aminohydrolase, ADA is a crucial enzyme in purine metabolism, as it regulates extracellular adenosine concentrations. Two isoenzymes are known—ADA1 and ADA2—with ADA1 functioning as an ectoenzyme anchored to the cell surface via P1 receptors [25,26].

We evaluated ADA gene expression in the aorta of mice subjected to the AKI model with and without IS treatment. No significant differences in ADA mRNA levels were observed in the aortas of mice exposed only to IR injury (Figure S3, Supplementary Materials). In contrast, ADA gene expression was significantly increased in the aorta of IR mice subjected to the IR + IS protocol (Figure 4D). These results indicate that ischemia, combined with saline or indoxyl sulfate exposure, induces upregulation of ADA, suggesting an important role for this enzyme in regulating purinergic signaling in the aorta. Furthermore, adenosine degradation may occur as a compensatory mechanism to balance extracellular levels. However, ADA gene expression was reduced in IR + IS animals compared with the IR + Saline group, possibly indicating decreased adenosine degradation (Figure 4D).

### 2.5. Circulating Inflammatory Cytokines Are Altered in Response to Renal IR Injury

**Figure 5:**
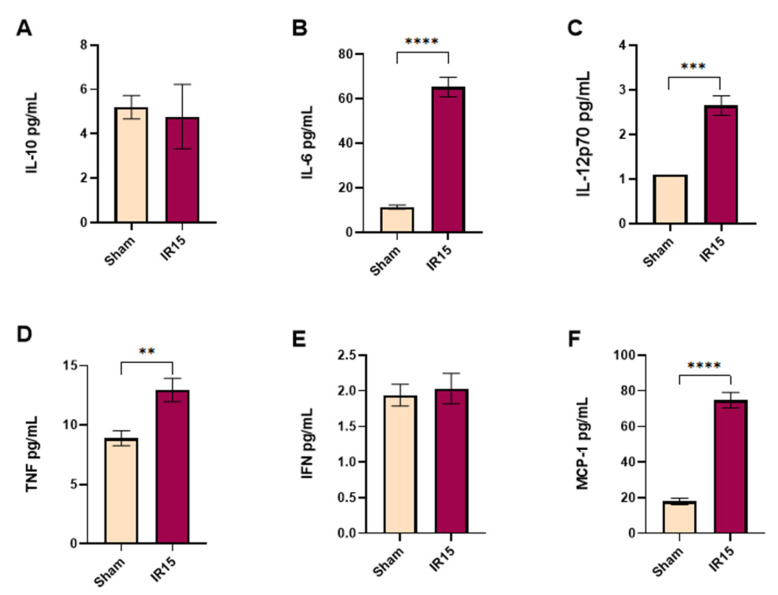
Plasma pro- and anti-inflammatory cytokine levels IL-10 (**A**), IL-6 (**B**), IL-12 (**C**), TNF-α (**D**), IFN-γ (**E**), and MCP-1 (**F**) in the plasma of animals of Sham and IR15 groups (n = 5 animals per group) after renal IR injury (** *p* < 0.01, *** *p* < 0.001, **** *p* < 0.0001).

Plasma inflammatory cytokines were quantified by flow cytometry. Changes were observed in IL-6 (Figure 5B), IL-12 (Figure 5C), TNF-α (Figure 5D), and MCP-1 (Figure 5F) levels, whereas no alterations were detected in IL-10 (Figure 5A) or IFN-γ (Figure 5E).

## 3. Discussion

Acute kidney injury (AKI) resulting from renal failure is associated with high morbidity and mortality and represents a critical event that occurs when an organ experiences reduced blood flow followed by reperfusion [27]. Reduced perfusion (ischemia) diminishes oxygen supply and disrupts renal cellular metabolism due to impaired ATP synthesis [27,28]. Restoration of blood flow during reperfusion reestablishes aerobic metabolism and pH balance in the kidney, but also promotes the formation of reactive oxygen species (ROS), which can damage functional cellular components and induce cell death by necrosis or apoptosis [27,28]. Additionally, renal hypoxia contributes to fibrosis characterized by tubular cell atrophy and extracellular matrix accumulation [28,29].

Studies employing the unilateral ischemia–reperfusion (IR) model have reported compensatory hypertrophy of the contralateral kidney after IR surgery, with increased mass in the non-ischemic kidney [19,30], corroborating the findings of the present study (Figure S1, Supplementary Materials). Moreover, indoxyl sulfate (IS) did not induce morphometric alterations in target organs, suggesting that during the evaluated period, the contralateral right kidney was able to compensate for renal function and eliminate excess IS (Figure S1, Supplementary Materials).

There is ongoing debate regarding the strategies used to diagnose AKI, as current methods exhibit low sensitivity and specificity. Although urea and creatinine have long served as conventional biomarkers for assessing renal function, their diagnostic utility is limited. For instance, variability in baseline serum creatinine levels can lead to false-positive results and confusion with other renal conditions such as chronic kidney disease (CKD) [31]. Furthermore, elevated creatinine concentration is not considered a sensitive marker of structural kidney injury, and together with urea, fails to accurately reflect the glomerular filtration rate (GFR). For a substance to serve as an ideal GFR marker, it must be excreted exclusively by the kidneys, not reabsorbed, and unaffected by extrarenal factors—criteria not fully met by current markers [22,32]. Therefore, the results presented here reinforce the limitations of using plasma urea and creatinine levels as indicators of renal function (Figure S2, Supplementary Materials). In contrast, neutrophil gelatinase-associated lipocalin (NGAL) and cystatin C have been recommended as more reliable alternatives for AKI detection and have shown promising results in clinical studies [33].

Despite ongoing debate regarding the validity of urea and creatinine as reliable markers for AKI, we also quantified plasma levels of other uremic toxins, including indoxyl sulfate (IS), p-cresyl sulfate (PCS), and indoleacetic acid (IAA). Increased plasma concentrations of IS and PCS were observed in the IR15 group compared with the Sham control, suggesting impaired renal filtration and accumulation of these toxins (Figure 1). Elevated levels of uremic toxins may indicate compromised renal function. Notably, high concentrations of IS have been shown to stimulate T cells in vitro and upregulate immunoregulatory genes such as CD39, which catalyzes the conversion of ATP to ADP and AMP [34]. The increase in this ectonucleotidase aligns with previous findings from our group demonstrating enhanced ATP hydrolysis after 15 days of reperfusion, accompanied by increased gene expression of CD39 (NTPDase1) [19].

We recently reported that AKI induced by IR increases the gene expression of purinergic receptors P2Y1, P2Y2, P2Y6, and P2X4, as well as ectonucleotidases NTPDase1, NTPDase2, and ecto-5′-nucleotidase, together with elevated ATP and AMP hydrolysis in the aorta of mice—potentially leading to greater adenosine production [19]. Building upon these findings, the present study evaluated the adenosinergic axis in response to the AKI models investigated. Several authors have reported that adenosine acts as a protective molecule against tissue injury in inflamed or ischemic environments [35]. Moreover, hypoxia has been shown to upregulate ectoenzymes that metabolize adenine nucleotides, thereby increasing adenosine production to restore homeostasis following inflammatory stress [36].

Overall, we observed increased transcript levels of all adenosine receptors (Figure 2 and Figure 3). The activation and modulation of these receptors are of great importance, as numerous studies have demonstrated their therapeutic potential in cardiovascular disorders [37]. These receptors also play protective roles against ischemic damage and contribute to anti-inflammatory responses [35].

The adenosine A1 receptor is widely recognized as a key protective mediator against IR injury in cardiovascular, renal, and other tissues, primarily acting through G-protein-coupled, anti-apoptotic, and antioxidant signaling pathways. Its activation before or during ischemia limits necrosis and enhances tissue functional recovery [38]. Under hypoxic conditions, adenosine released into the extracellular space activates endothelial A1 receptors, stimulating nitric oxide (NO) synthesis and release, promoting vasodilation, and reducing damage associated with excessive ROS and oxidative stress [39,40]. Thus, our results suggest that inflammation and endothelial dysfunction induced by IR and/or IS (Figure 2A and Figure 3A) may alter A1 receptor gene expression, indicating a possible protective role for this receptor in the vascular context of AKI-associated inflammation.

The adenosine A2A receptor appears to play a pivotal role in mitigating damage caused by renal failure. In addition to protecting the kidneys from ischemia and reperfusion injury, it can reduce the expression of pro-inflammatory cytokines (TGF-β, IL-1β, IL-1ra, and IL-6) in the kidneys of wild-type (WT) mice, but not in A2A-knockout animals [41]. Consistent with these renal findings, our results demonstrate activation of A2A receptors in the aortas of IR15 animals, both with and without IS administration (Figure 2B and Figure 3B). A2A activation reduces IR-induced rolling, adhesion, and transmigration of inflammatory cells [42], thereby limiting their recruitment to injured tissues and decreasing endothelial damage. Interestingly, we observed increased A2A gene expression in the aortas of IR + IS animals compared with IR + Saline animals (Figure 3B), suggesting enhanced vascular protection in animals subjected to both IR injury and IS treatment.

Importantly, activation of the A2B receptor has been reported to attenuate inflammatory responses in tissues distant from the ischemic site. This receptor reduces damage following renal IR, alleviating lung injury and improving oxygenation [43]. In the aortas of animals subjected to IR followed by 15 days of reperfusion, a significant increase in A2B transcript levels was observed (Figure 2C). Given that this receptor limits inflammation, prevents vascular leakage, promotes angiogenesis (via VEGF and eNOS), and reduces vascular lesion formation [44], our results corroborate the protective role of adenosine receptors in the context of AKI.

It is noteworthy that A2B has the lowest affinity for adenosine, requiring higher agonist concentrations for activation [26,45]. The A2B gene promoter contains a hypoxia-response element (HRE), a specific DNA sequence within promoter or enhancer regions that serves as a binding site for the hypoxia-inducible factor (HIF) transcription factor. Under hypoxic conditions, HIF-1α binds to the HRE and directly activates A2B gene transcription [46,47]. However, IS has been shown to inhibit nuclear accumulation of HIF-1α in hypoxia-exposed cells and to suppress hypoxia-induced HIF-1α elevation in endothelial progenitor cells [48,49]. Therefore, the decreased A2B gene expression observed in the Sham + IS group may reflect an inhibitory effect of IS on the HIF-1α/HRE pathway, which is essential for A2B transcriptional activation (Figure 3C).

The A3 receptor subtype also exhibited increased transcript levels in response to ischemia–reperfusion injury (Figure 2D), indicating generalized activation of the adenosinergic axis in the aorta during AKI. This receptor is crucial for restoring vascular reactivity and confers cardioprotection during ischemic events [50]. No significant differences were observed among experimental groups when animals were subjected to IR combined with IS administration (Figure 3D).

Ectonucleotidases are key purinergic enzymes responsible for degrading ATP to adenosine. In cardiac transplantation, NTPDase1 and ecto-5′-nucleotidase mediate protection against cardiac and renal failure [51]. In the present study, we observed increased expression of vascular ectonucleotidases NTPDase1, NTPDase2, and ecto-5′-nucleotidase in response to ischemic insult and IS administration, modulating purinergic signaling dynamics in the aorta (Figure 4). These findings align with previous evidence showing that adenosine, produced through the coordinated activity of NTPDase1 and ecto-5′-nucleotidase, is fundamental for tissue protection against hypoxic and ischemic injury [52].

Adenosine deaminase (ADA) is another key enzyme involved in the catabolism of adenosine to inosine [24]. A significant increase in ADA gene expression was observed in the aortas of animals from the IR + Saline and IR + IS groups compared with their respective Sham + Saline and Sham + IS controls (Figure 4D). These findings indicate that ischemia, with or without IS administration, induces upregulation of ADA, suggesting an important role for this enzyme in regulating purinergic signaling in the aorta of mice. Furthermore, adenosine degradation to inosine may occur as a compensatory mechanism to maintain extracellular homeostasis. However, ADA gene expression was reduced in the IR + IS group compared with the IR + Saline group, possibly indicating that animals subjected to AKI induced by IR and IS exhibit a more intense inflammatory state, requiring higher adenosine levels and consequent receptor activation.

Acute kidney injury has been consistently associated with elevated systemic levels of cytokines and inflammatory mediators, contributing to inflammation and injury in extra-renal organs. Previous studies have demonstrated that cytokines such as IL-6 and IL-12 progressively increase after reperfusion, and that following a 60 min renal ischemic event with 36 h of reperfusion, more than 50 inflammation-related genes were altered in the kidneys and over 20 in lung tissue—highlighting the potential of AKI to affect distant organs after ischemic insult [53,54]. Moreover, genomic and transcriptomic analyses have revealed that intrarenal inflammation promotes a similar inflammatory gene expression profile between the kidneys and distant tissues, such as the lungs [54].

As shown in Figure 5, increased plasma levels of cytokines IL-6, IL-12, TNF-α, and MCP-1 were detected in IR15 animals. The elevation of circulating inflammatory molecules can trigger systemic consequences and modulate purinergic signaling in peripheral tissues. Previous studies have demonstrated that increased TNF-α and IL-1α levels activate the NF-κB pathway, which enhances A2A receptor transcription and subsequently promotes IL-10 production, leading to anti-inflammatory and protective effects [55]. Similar regulatory mechanisms have been described for other adenosinergic receptors, including TNF-α-induced upregulation of A3 gene expression and IL-6-mediated potentiation of neuronal A1 receptor expression and function [56,57].

Beyond receptor modulation, in vitro models have shown that inflammatory cytokines such as IL-6 and TNF-α can stimulate the release of extracellular vesicles enriched in CD39 and CD73 ectonucleotidases. These vesicles are capable of modulating purinergic signaling in tissues and regulating processes such as angiogenesis and vascular remodeling [58].

Taken together, our results indicate that AKI induces a systemic increase in inflammatory cytokines that can modulate the adenosinergic axis of purinergic signaling in vascular beds distant from the kidney, such as the aorta.

## 4. Materials and Methods

In some experiments, we exclusively employed a model of acute kidney injury (AKI) induced by unilateral ischemia–reperfusion (IR) in the left kidney. This model is widely used because it reproduces clinical conditions commonly observed in hospital settings, such as kidney injury resulting from surgery, sepsis, or trauma. Also, the unilateral 60 min ischemia protocol provided the best balance between reproducibility and animal survival, showing the lowest mortality rate while efficiently inducing AKI and systemic consequences, including cardiac molecular and electrical changes as already shown [30]. In other experiments, this model was combined with the administration of indoxyl sulfate (IS), a uremic toxin known to promote oxidative stress, inflammation, and vascular dysfunction, in order to evaluate its additional effects in the context of kidney injury. The experimental groups corresponding to each approach are described below.

### 4.1. Experimental Groups

The experimental protocols were developed in accordance with the Arouca Federal Law (2008) and approved by the Research Ethics Committee (CEUA) of the Federal University of ABC (CEUA protocols No. 1467010424, No. 7995100124, and No. 7284191124). C57BL/6 male mice, 6–8 weeks, weighing between 20 and 28 g, were kept in cages (one animal/cage) and subjected to a 12 h artificial light/dark cycle at a constant room temperature of 25 °C with water and mouse chow ad libitum. The animals were divided into seven experimental groups: Sham (control), IR8, IR15, Sham+Saline (control), Sham+IS, IR+Saline, and IR+IS.

**Figure 6:**
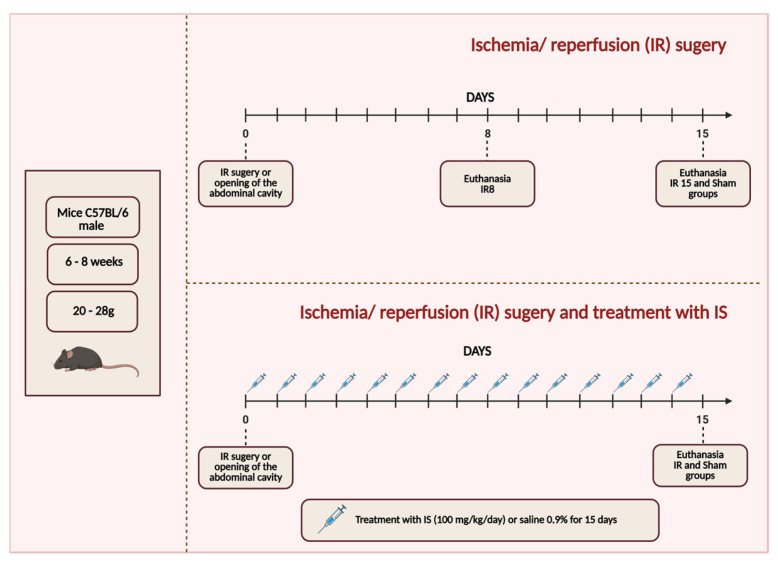
Experimental design. Created in BioRender. Furstenau, C. (2026) https://BioRender.com/hbuhe0f (accessed on 3 July 2026).

In the Sham, Sham+Saline, and Sham+IS groups, the animals underwent a laparotomy without occlusion of the left renal pedicle. The Sham+Saline and Sham+IS groups received injections of 0.9% saline solution or indoxyl sulfate (100 mg/kg/day) for 15 days. After 15 days, they were euthanized. The animals in the IR8 and IR15 day groups underwent laparotomy and left renal pedicle occlusion for 60 min, followed by removal of the vascular clip for organ reperfusion, and were euthanized after 8 or 15 days of reperfusion, respectively. Finally, in the IR+Saline and IR+IS groups, the animals underwent the same ischemia/reperfusion protocol described previously, followed by treatment with 0.9% saline solution or indoxyl sulfate (100 mg/kg/day) for 15 days. Euthanasia was performed at the end of this period. The experimental design is shown in Figure 6.

### 4.2. Euthanasia of Animals and Collection of Tissues and Biological Samples

Animals were anesthetized by intraperitoneal injection of a mixture containing 240 mg/kg ketamine and 30 mg/kg xylazine (Agribands do Brasil Ltda, Campinas-Paulina, Brazil). The abdominal cavities were opened, and the intestinal loops were displaced for venous blood puncture via the inferior vena cava, followed by the removal and weighing of the kidneys. For the extraction of the thoracic part of the aorta, as well as for the collection and weighing of the heart, the thoracic cavity was also opened. In addition, the right tibia of the mice was dissected and measured with calipers for use as a normalizer for morphometric measurements. For plasma collection, blood was collected and mixed with 0.5 M EDTA at a volume equivalent to the collected blood (1:10 ratio). Subsequently, the samples were centrifuged at 4 °C, 10 × 1000× *g* for 15 min.

### 4.3. Confirmation of the Experimental Model

To assess the effect of AKI, morphometric indices were obtained using heart weight (g), right and left kidney weight (g), and tibia length (mm). The ratio between heart weight (HW), left kidney weight (LKW), right kidney weight (RKW), and tibia length (TL) was evaluated in all experimental groups. Creatinine and urea levels were also measured in the plasma of animals from all experimental groups using diagnostic kits from LabTest Diagnóstica (Lagoa Santa, Brazil), according to the manufacturer’s instructions.

### 4.4. Uremic Toxins Dosage

From the plasma of Sham and IR15 animals, the total concentrations of uremic toxins IS, p-CS, and IAA were quantified by high-performance liquid chromatography (HPLC) with fluorescence detection. Ultrafiltered plasma was injected into an HPLC system, and separation was achieved using a 150 × 4.6 mm, 5 μm Luna C8 column (Phenomenex, Torrence, CA, USA), eluted with 50 mM ammonium formate, pH 3.0, and methanol. Fluorescence wavelengths ranged: λ exc = 280 nm and λ em = 383 nm for IS and IAA, λ exc = 265 nm and λ em = 290 nm for p-CS [59].

### 4.5. Gene Expression Analysis by Real-Time PCR (qPCR)

Total RNA from the aortas of mice from all experimental groups was obtained using the Trizol^®^ reagent (GIBCO – Life Technologies, Grand Island, NY, USA), followed by integrity analysis. cDNA was prepared using reverse transcription with the Proto Script kit (New England BioLabs^®^, Ipswich, MA, USA). The cDNA was then amplified by polymerase chain reaction (PCR) using specific primer sequences (Table S1, Supplementary Materials) for the adenosine receptor genes Adora1, Adora2a, Adora2b, Adora3, as well as the ectonucleotidases Entpd1 (NTPDase1), Entpd2 (NTPDase2), Nt5e (ecto-5’-nucleotidase), and Ada (adenosine deaminase). Real-time PCR was performed using the SYBR Green PCR mixture (Qiagen, Hilden, Germany) in a thermocycler Rotor-Gene Q (Qiagen, Hilden, Germany). The reported sample size (N) refers to biological replicates (individual animals), while each qPCR reaction was performed in technical duplicate. Gene expression was analyzed using the 2−ΔΔCt method, with the Sham or Sham+Saline group serving as calibrators, and the data were normalized with the β-actin gene.

### 4.6. Quantification of Cytokines

To quantify levels of inflammatory cytokines in mouse samples, the BD™ Cytometric Bead Array (CBA) Mouse Inflammation Kit (Catalog No. 552364, BD Biosciences, San Diego, CA, USA) was used according to the manufacturer’s instructions. This multiplex assay allows the simultaneous detection of six soluble analytes—Interleukin-6 (IL-6), Interleukin-10 (IL-10), Monocyte Chemoattractant Protein-1 (MCP-1), Interferon-γ (IFN-γ), Tumor Necrosis Factor (TNF-α), and Interleukin-12p70 (IL-12p70)—in a single sample, using flow cytometry. A total of 5 mouse plasma samples per experimental group were used for cytokine quantification. Cytokine concentrations were calculated based on standard curves and expressed in picograms per milliliter (pg/mL). Cytometric analysis was performed using the Cytoflex LX™ flow cytometer (Beckman Coulter, Brea, CA, USA), and the data were analyzed with FlowJo 10.0.8 (Ashland, OR, USA).

### 4.7. Analysis of Results

Data is presented as means ± standard error of the means (∆±SEM) for each measurement across all groups studied. Data distribution was assessed using the Shapiro–Wilk normality test. For normally distributed data, comparisons between groups were performed using one-way or two-way analysis of variance (ANOVA) (depending on the group analyzed), followed by Tukey’s post hoc test; for groups that did not show a normal distribution, Dunn’s post hoc test was used for multiple comparisons. Differences were considered statistically significant when p < 0.05. GraphPad Prism software (Boston, MA, USA) (version 8.0.1 for Windows) was used for statistical data analysis and graph generation. Figures and graphs were created using BioRender (Toronto, ON, Canadá) (https://BioRender.com/qmvlmd5).

## 5. Conclusions

This study investigated the impact of acute kidney injury (AKI) induced by renal ischemia–reperfusion (IR), with or without indoxyl sulfate (IS) treatment, on the adenosinergic axis of purinergic signaling in the aorta of mice. Validation of the experimental model confirmed atrophy of the ischemic kidney and compensatory hypertrophy of the contralateral kidney, together with increased levels of plasma protein-bound uremic toxins, indicating impaired renal function even in the absence of changes in classical markers such as urea and creatinine. Gene expression analysis revealed a significant increase in transcripts of all P1 receptors (A1, A2A, A2B, and A3) at 15 days of reperfusion, demonstrating that AKI can markedly modulate the purinergic profile in a vascular bed distant from the site of injury. This modulation appears to occur in parallel with the previously described enhancement of CD39-dependent adenosinergic activity after IR, suggesting a shift of the purinergic pathway toward a more adenosinergic state, potentially associated with inflammatory control and tissue protection mechanisms. Similarly, when combined with IS administration, increased gene expression of ectonucleotidases and A1 and A2A receptors was observed, consistent with the results obtained for the IR15 group. These findings expand current understanding of the extra-renal effects of AKI and IS, supporting the hypothesis that P1 receptors may act as mediators of compensatory mechanisms that preserve vascular homeostasis. Further studies employing more sensitive approaches for purine quantification, functional analyses of vascular tone, and genetic models are necessary to elucidate the direct contribution of P1 receptors to the vascular alterations associated with AKI. Finally, these results may contribute to the discovery of new biomarkers and therapeutic targets based on purinergic signaling for the diagnosis and treatment of vascular complications associated with AKI in the future.

## Data Availability

No new data were created or analyzed in this study. Data sharing is not applicable to this article.

## Supplementary Materials

The following supporting information can be downloaded at: https://www.mdpi.com/article/10.3390/ijms27146296/s1.

## Abbreviations

AKI	Acute kidney injury
IR	Ischemia-reperfusion
UT	Uremic toxins
IS	Indoxyl sulfate
ICU	Intensive care unit
KDIGO	Kidney Disease Improving Global
VSMC	Vascular smooth muscle cells
ROS	Reactive oxygen species
CKD	Chronic kidney disease
DAMP	Damage-associated molecular pattern
ENPPs	Ectonucleotide pyrophosphate/phosphodiesterases
ENTPDases	Ectonucleoside triphosphate diphosphohydrolases
CEUA	Research ethics committee
HW	Heart weight
LKW	Left kidney weight
RKW	Right kidney weight
TL	Tibia length
HPLC	High-performance liquid chromatography
PCR	Polymerase chain reaction
CBA	Cytometric bead array
IL-6	Interleukin-6
IL-10	Interleukin-10
MCP-1	Monocyte Chemoattractant Protein-1
IFN-γ	Interferon-γ
TNF-α	Tumor necrosis factor
IL-12p70	Interleukin-12p70
ANOVA	Analysis of variance
GFR	Glomerular filtration rate
PCS	P-cresyl sulfate
IAA	Indoleacetic acid
VEGF	Vascular endothelial growth factor
eNOS	Endothelial nitric oxide synthase
HRE	Hypoxia response
HIF	Hypoxia-inducible factor
ADA	Adenosine deaminase
