# Supplementary material for: Renal Ischemia–Reperfusion and Uremic Toxins Modulate the Aortic Adenosinergic Axis in Acute Kidney Injury
Source: Int J Mol Sci. 2026 Jul 15;27(14):6296. doi: 10.3390/ijms27146296 (PMC13410169; doi:10.3390/ijms27146296)
Supplement: Supplementary file 1 [file ijms-27-06296-s001.zip › ijms-4387306-supplementary.pdf]

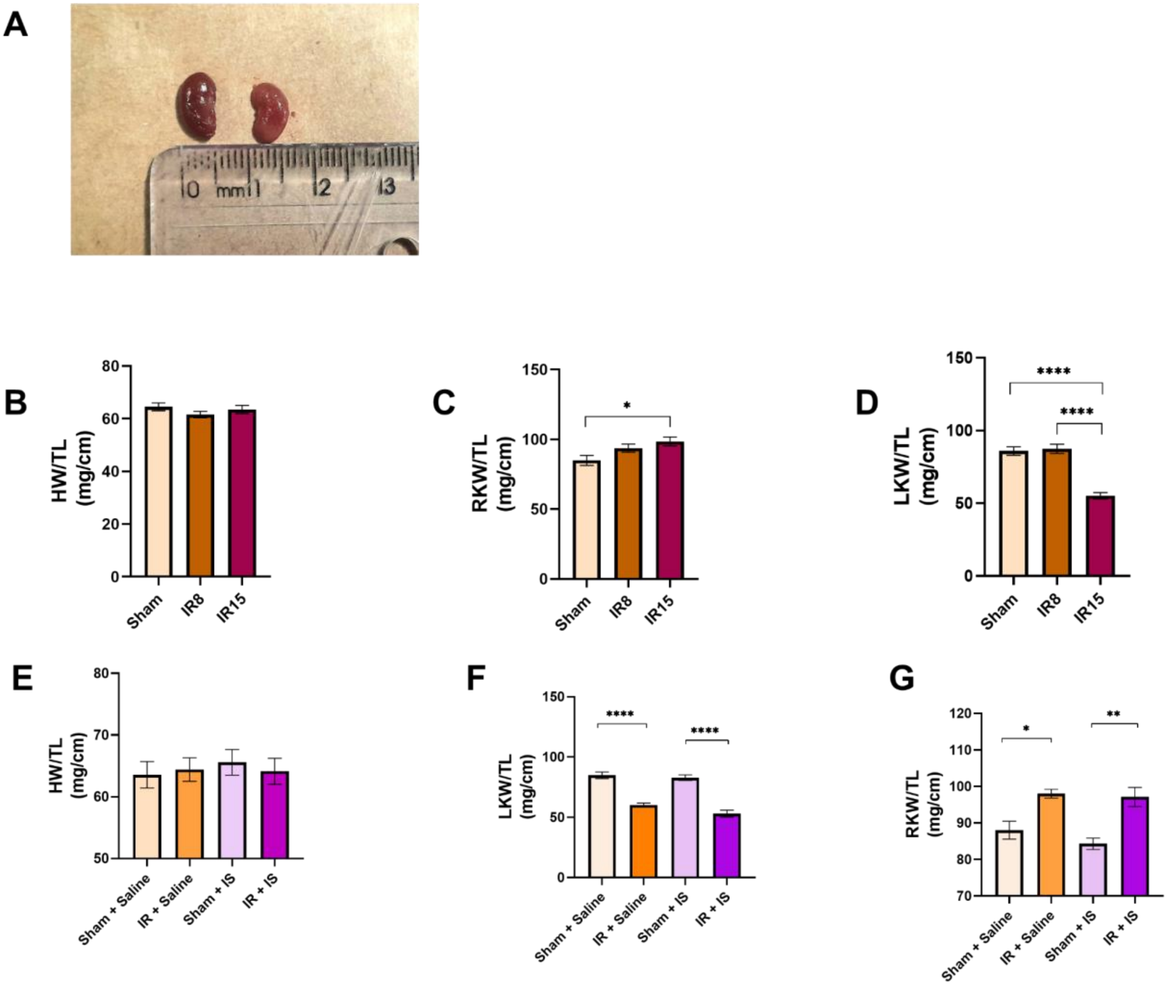

Figure S1. Confirmation of AKI models by IR and IR+IS. (A) Representative picture of the injured left kidney (LK) and the contralateral right kidney (RK) after 15 days of reperfusion. Cardiac (B, E) and right (C, F) and left (D, G) renal mass indices by tibial length (mg/cm) of different experimental groups (N = 10 animals per group). HW= heart weight; RKW = right kidney weight; LKW = left kidney weight; TL = tibia's length. (****$p<0.0001$, **$p < 0.01$, *$p < 0.05$).

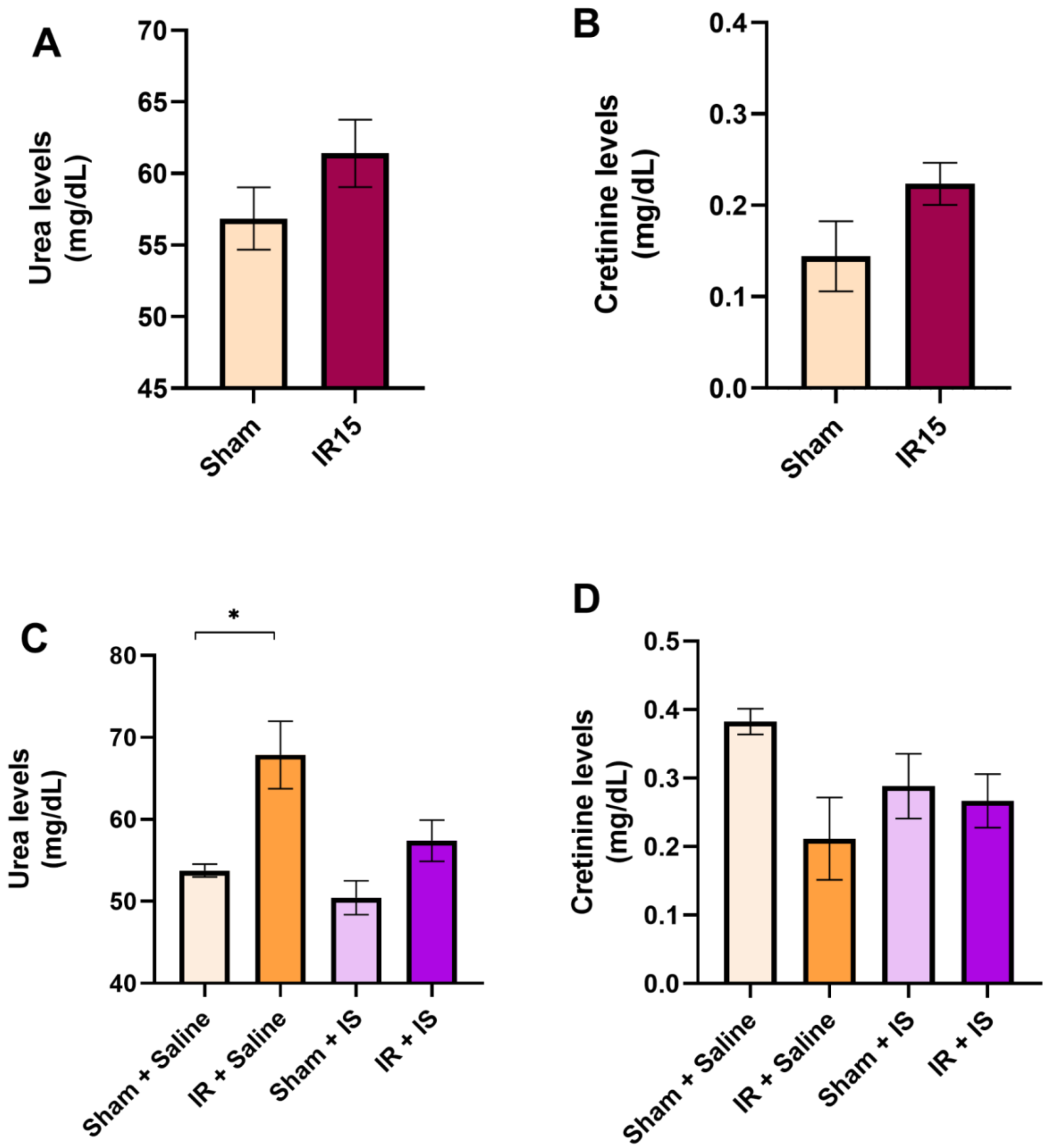

Figure S2. Biochemical analysis of renal function. Plasma urea levels (mg/dL) in Sham and IR15 animals (A) and in Sham+Saline, IR+saline, Sham+IS and IR+IS animals (C). Plasma creatinine levels (mg/dL) of Sham and IR15 animals (B) and in Sham+Saline, IR+saline, Sham+IS and IR+IS animals (D) (N = 5 animals per group) ($*p < 0.05$).

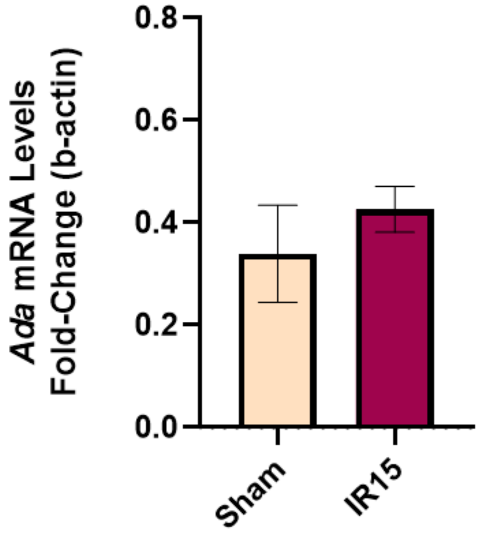

Figure S3. Analysis of adenosine deaminase (ADA) gene expression. Relative gene expression of ADA in aortas from animals of the Sham e IR15 groups (N = 4 animals per group). β-actin was the normalizing gene.

Table S1. The sequence of primers used for the genes of interest

Gene	Sense sequence	Anti-sense sequence
Actb	GGGAAATCGTGCGTGACATC	GCCACAGGATTCCATACCCAA
Adora1	GGTACAAGACAGTGGTGACTCAG	CTATCCAGGCTTGTTCCACC
Adora2a	CCACTCCGGTACAATGGCT	GCAGGTCTTTGTGGAGTTCTCA
Adora2b	CGTCCCGCTCAGGTATAAAG	CCAGTTCTGTGCAGTTGCTG
Adora3	CCACGTTCTATTTCATTGTCTCC	AGGCAGGCATAGAAGTGCAT
Entpd1	GCAGCAGTTAGAGGAATG	GCACCGATTTCATCTGTT
Entpd2	GGCCAGCATTACCGTGTCTA	GGTGGAAGCGATGGATCTGG
Nt5e	TCCTCTCAAATCCAGGGACA	CAAAACCAGAGTGCCCCA
Ada	CGGTTGTTCGCTTCAAGAAT	TCAGTGAAGCCCATGTCTTTC

Actb - gene encoding beta-actin; Adora1 - gene encoding purinergic receptor A1; Adora2a - gene encoding purinergic receptor A2A; Adora2b - gene encoding purinergic receptor A2B; Adora3 - gene encoding purinergic receptor A3; Entpd1 - gene encoding ectonucleoside triphosphate diphosphohydrolase 1; Entpd2 - gene encoding ectonucleoside triphosphate diphosphohydrolase 2; Nt5e - gene encoding ecto-5′-nucleotidase; Ada - gene encoding adenosine deaminase.

ATP and adenosine dosage

ATP and adenosine levels were evaluated in aortas from Sham, IR8, and IR15 animals using commercial kits from Sigma Aldrich® (Merck KGaA, Darmstadt, Germany), specifically MAK190 for ATP and MAK433 for adenosine, according to the manufacturer's instructions.

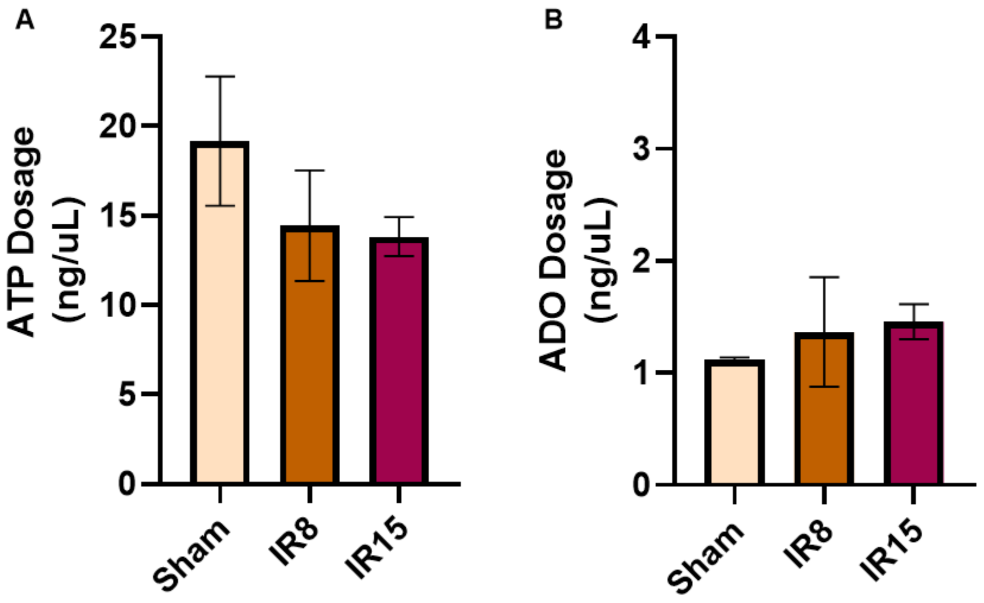

Figure S4. Measurement of ATP (A) and adenosine (B) levels, respectively, in the aorta and plasma of mice from the Sham, IR8, and IR15 groups (N = 5 animals per group).

ATP and ADO dosage

ATP is a pro-inflammatory molecule in the extracellular context, as several studies have demonstrated that ATP acts as an inflammatory DAMP (damage-associated molecular pattern), recruiting innate immune cells, promoting the production of inflammatory mediators, and stimulating the release of pro-inflammatory cytokines, potentially leading to pathological conditions. Moreover, increased vascular injury has been observed under hypoxic conditions and systemic inflammation in CD39- and CD73-knockout mice (Dou et al., 2018), enzymes that together catalyze the breakdown of ATP into adenosine.

Given that ischemia–reperfusion (IR)-induced acute kidney injury (AKI) triggers a systemic inflammatory response, we hypothesized that this inflammation could affect the CD39 adenosinergic axis, thereby altering ATP and adenosine levels in the aorta. Therefore, ATP and adenosine levels were measured respectively in the aorta and plasma of mice subjected to IR-

induced AKI (Figure S4), with each analyte assessed in a distinct sample type due to assay kit limitations.

Although increased transcript levels of NTPDase1, NTPDase2, and ecto-5′-nucleotidase, as well as enhanced ATP and AMP hydrolysis in the aorta of IR15 mice, have been reported (Stabile et al., 2024), no significant differences were found in aortic ATP levels or plasma adenosine levels among the experimental groups in the present study.

Several considerations are important when interpreting these findings. First, the sample size may have been insufficient to detect significant changes. In addition, adenosine was measured in plasma rather than in the aorta, where P1 receptor gene expression was increased; therefore, direct inferences about adenosine levels within the vessel wall cannot be made. Finally, although the fluorimetric kits used were sensitive, they may not represent the most suitable approach for quantifying these molecules, as discussed below.

Multiple studies have attempted to measure ATP and adenosine concentrations in plasma and tissues, yet consensus remains elusive because both molecules exhibit extremely short half-lives. Reports indicate that adenosine's half-life can be less than 1.5 s (Lerman et al., 1991), whereas ATP's half-life in arterial blood may be under 1 s (Mortensen et al., 2011). In the extracellular milieu, ATP and ADP are rapidly hydrolyzed by ectonucleotidases such as NTPDase, generating AMP, which is subsequently converted to adenosine through the action of ecto-5′-nucleotidase. Extracellular adenosine is then swiftly deaminated to inosine by adenosine deaminase (ADA), another enzyme of the purinergic pathway (Dwyer et al., 2020). Therefore, additional methodological approaches are required to more accurately determine ATP and adenosine concentrations in the mouse aorta.
